# The Impact of a Lockdown for the COVID-19 Pandemic on Seasonal HbA1c Variation in Patients with Type 2 Diabetes

**DOI:** 10.3390/life13030763

**Published:** 2023-03-11

**Authors:** Yu-Cheng Cheng, Yu-Hsuan Li, Hsiu-Chen Liu, Chiann-Yi Hsu, Wan-Jen Chang, I-Te Lee, Chin-Li Lu

**Affiliations:** 1 Division of Endocrinology and Metabolism, Department of Internal Medicine, Taichung Veterans General Hospital, Taichung 40705, Taiwan; 2 School of Medicine, National Yang Ming Chiao Tung University, Taipei 11221, Taiwan; 3 Institute of Biomedical Sciences, National Chung Hsing University, Taichung 40227, Taiwan; 4 Department of Computer Science & Information Engineering, National Taiwan University, Taipei 10617, Taiwan; 5 Department of Nursing, Taichung Veterans General Hospital, Taichung 40705, Taiwan; 6 Biostatistics Task Force, Taichung Veterans General Hospital, Taichung 40705, Taiwan; 7 School of Medicine, Chung Shan Medical University, Taichung 40201, Taiwan; 8 Graduate Institute of Food Safety, College of Agriculture and Natural Resources, National Chung Hsing University, Taichung 40227, Taiwan; 9 Department of Food Science and Biotechnology, College of Agriculture and Natural Resources, National Chung Hsing University, Taichung 40227, Taiwan; 10 Department of Post-Baccalaureate Medicine, College of Medicine, National Chung Hsing University, Taichung 40227, Taiwan

**Keywords:** COVID-19, glycated hemoglobin, lockdown, type 2 diabetes mellitus, season, variation

## Abstract

Glycemic control in patients with type 2 diabetes may be disrupted due to restricted medical service access and lifestyle changes during COVID-19 lockdown period. This retrospective cohort study examined changes of HbA1c levels in adults with type 2 diabetes 12 weeks before and after May 19 in 2021, the date that COVID-19 lockdown began in Taiwan. The mean levels of HbA1c-after were significantly lower than HbA1c-before in 2019 (7.27 ± 1.27% vs 7.43 ± 1.38%, *p* < 0.001), 2020 (7.27 ± 1.28% vs 7.37 ± 1.34%, *p* < 0.001), and 2021 (7.03 ± 1.22% vs 7.17 ± 1.29%, *p* < 0.001). Considering the seasonal variation of HbA1c, ΔHbA1c values (HbA1c-after minus HbA1c-before) in 2020 (with sporadic COVID-19 cases and no lockdown) were not significantly different from 2021 (regression coefficient [95% CI] = 0.01% [−0.02%, 0.03%]), while seasonal HbA1c variation in 2019 (no COVID-19) was significantly more obvious than in 2021 (−0.05% [−0.07, −0.02%]). In conclusion, HbA1c level did not deteriorate after a lockdown measure during the COVID-19 pandemic in Taiwan. However, the absolute seasonal reduction in HbA1c was slightly less during the COVID-19 pandemic compared with the year without COVID-19.

## 1. Introduction

The spread of coronavirus disease 2019 (COVID-19) was declared a global pandemic and became an international health crisis [1]. A number of lockdown measures were implemented to slow the spread of the virus worldwide. However, social distancing and other restrictions might lead to a reduction in clinical accessibility during lockdown measures [2]. The World Health Organization reported that diabetic treatment was partially or completely disrupted in 49% of the 155 countries surveyed in May 2020 [3]. Moreover, among patients with type 2 diabetes mellitus (DM), an increase in carbohydrate intake and a decrease in physical activity have been reported following COVID-19 confinement [4,5]. Adverse lifestyle changes may promote weight gain and glycated hemoglobin (HbA1c) levels [6,7,8]. In particular, poor glucose control preceding COVID-19 infection increases the risk of adverse outcomes [9]. Therefore, it is necessary to evaluate the impact of lockdown measures during the COVID-19 pandemic.

The impact of the COVID-19 lockdown on glucose control has been widely studied in type 2 DM. Unlike the study results in type 1 DM, which consistently reported an improvement in glycemic control [10], the findings in type 2 DM were heterogeneous [11,12,13]. Silverii et al. [11] showed that lockdown measures had no significant effect on HbA1c levels in a meta-analysis of observational studies in a population with type 2 DM. However, some other studies found that a COVID-19 lockdown was associated with a significant increase in HbA1c [13] and fasting glucose levels [13,14] in patients with type 2 DM. Another two more recent meta-analyses reported a reduction in mean glucose levels [15] and an insignificant change in HbA1c levels [14] after lockdown; nevertheless, the two studies pooled the results from both type 1 and type 2 DM, mostly from type 1 DM.

As interpreting the observed changes in glycemic control during COVID-19 lockdown, seasonal variation should be taken into consideration [10]. Many studies have shown that glucose levels are higher in cold seasons than in warm seasons [16,17,18]. It has been reported that winter was significantly associated with increased admission rates of both diabetic ketoacidosis and hyperglycemic hyperosmolar state [19]. However, most previous studies simply compared the glucose levels before and after (or during) the lockdown in the same year [11,12,13,14]. The before-and-after comparison without referenced years restricts the feasibility to clarifying whether the observed changes in glucose levels were resulted from lockdown measures or seasonal variation. In addition, most previous studies of glycemic control following the implementation of COVID-19 lockdown measures had relatively small sample sizes and limited the statistical power to detect the changes in glucose markers [11,12,13,14]. In Taiwan, an extremely rigorous border control has been in force since January 2020 [20]. There were only sporadic cases and locally small-scale outbreaks of COVID-19 in 2019 and 2020, respectively. Due to a surge of confirmed COVID-19 cases in mid-May 2021, the nation-wide COVID-19 alert was raised to level 3 (Lv3) from level 2 (Lv2) from 19 May 2021, until 27 July 2021, according to the epidemic warning standards and guidelines announced by the Taiwan Centers for Disease Control. The measures implemented under the COVID-19 level 3 alert (Lv3 alert) included gathering restrictions, closing of businesses and public areas, social distancing, and avoiding non-essential travel from one’s home [21,22]. This Lv3 alert lasted for 69 days until 27 July 2021. After that, the alert level was downgraded to Lv2, which still recommended social distancing but allowed leisure activities to occur in public places.

To explicitly investigate the impact of the COVID-19 lockdown on glycemic control in Taiwan with appropriate consideration of seasonal effect on glucose levels, we conducted a retrospective cohort study in adult patients with type 2 DM in Taiwan, which compared the seasonal changes in HbA1c levels before and after the Lv3 alert over three years, from the year without COVID-19 (2019), the year with COVID-19 but without Lv3 alert (2020), to the year with COVID-19 and Lv3 alert (2021).

## 2. Materials and Methods

### 2.1. Study Design and Patients

**Figure 1:**
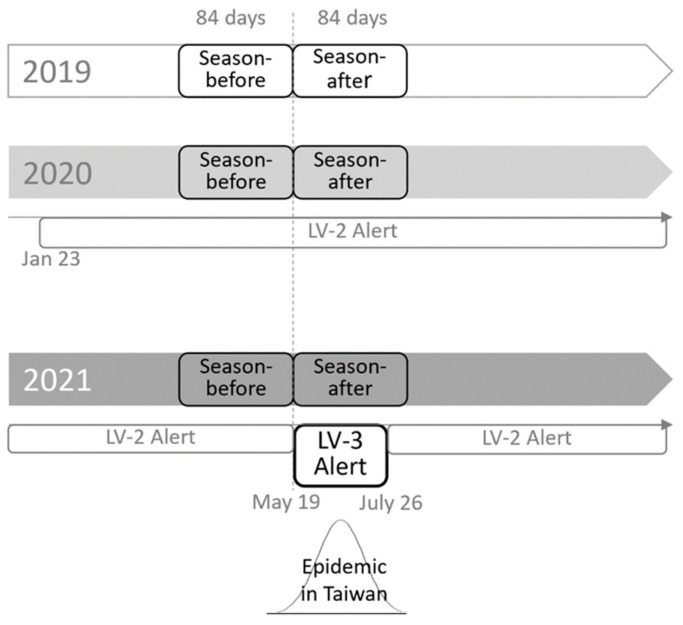
Diagram of study design.

This retrospective cohort study was conducted at Taichung Veterans General Hospital (VGH) between 2019 and 2021. The Lv3 alert was issued on 19 May for the COVID-19 epidemic. Based on the different observation years, we enrolled patients into three study groups, which corresponded to three distinct scenarios of COVID-19 outbreaks: no COVID-19 cases (cohort-2019), only small-scale and local COVID-19 outbreaks with an Lv2 alert in Taiwan (cohort-2020), and with a COVID-19 epidemic and Lv3 alert in Taiwan (cohort-2021). The longest duration for refilling prescriptions was 12 weeks in Taichung VGH; therefore, the study period in each year was divided into season-before and season-after according to the start date of the Lv3 alert on 19 May 2021. The season-before was defined as the 12-week period before 19 May (i.e., between 14 February and 18 May), and the season-after was defined as the 12-week period following 19 May (i.e., between 19 May and 10 August). The study design is illustrated in Figure 1.

The inclusion criteria were as follows: (1) outpatients with a diagnostic International Classification of Diseases (ICD) code record of 250 for the ICD 9th version or E11–E13 for the ICD 10th version before 14 February in the observation year and (2) having at least one HbA1c record in both the season-before and season-after, respectively, in the observation year. The exclusion criteria were as follows: (1) a history of hospitalization between 1 January and 10 August during the observation year; (2) age < 20 years; (3) type 1 DM; (4) other types of DM, including pancreatic, hepatic, and secondary diabetes due to endocrine diseases; (5) gestational diabetes; (6) pregnancy at the time of HbA1c examination; (7) a history of anemia, which was defined as ICD (9th version) of 280–285 or ICD (10th version) of D50-D64; (8) a history of steroid use between 1 January and 19 May in each observation year; and (9) a history of participating in Ramaḍān during the study season. Anonymous demographic characteristics and laboratory data were obtained from the Clinical Informatics Research and Development Center of Taichung VGH after delinking the identification information. The study protocol was approved by the Institutional Review Board of the Taichung VGH in Taiwan, with a waiver for obtaining informed consent.

### 2.2. Measurements

We collected only the latest records of multiple HbA1c measurements during each study season in the observation year. Therefore, each patient had paired HbA1c values (HbA1c-before and HbA1c-after) in the observation year. The ΔHbA1c was defined as (HbA1c-after)−(HbA1c-before) in the same observation year. The other baseline characteristics were retrieved within the baseline period between 1 January and 19 May in each observation year, including age, sex, height, body weight, systolic blood pressure (SBP), diastolic blood pressure (DBP), heart rate (HR), serum levels of glutamic pyruvic transaminase (GPT), creatinine, low-density lipoprotein (LDL) cholesterol, and triglycerides (TG). Among multiple measurements during the baseline period, only the latest data were collected. The use of antidiabetic and antihypertensive drugs was defined as medication prescribed between 1 January and 19 May in each observation year.

In the clinical practice of diabetes management, blood samples for biochemical analyses were collected the morning after an overnight fast. HbA1c levels were measured using cation-exchange high-performance liquid chromatography (National Glycohemoglobin Standardization Program, G8, TOSOH, Tokyo, Japan). Biochemical analyses were performed using a photometric enzymatic method with a chemical analyzer (Hitachi 7600, Tokyo, Japan). Body mass index (BMI) was calculated as the weight (kg)/(height (m))^2^. The estimated glomerular filtration rate (eGFR) was calculated using the modification of diet in renal disease equation, as follows: 186 × (serum creatinine)^−1.154^ × (age)^−0.203^ (×0.742 if female) [23].

Hypertension was defined as the use of any antihypertensive drugs (ICD 9th version of 401–409 or ICD 10th version of I10–I15). Chronic kidney disease was defined as an eGFR < 60 mL/min/1.73 m^2^. Hypercholesterolemia was defined as an LDL level ≥ 100 mg/dL, and hypertriglyceridemia was defined as a TG level ≥ 150 mg/dL according to the reference target goal [24,25].

### 2.3. Statistical Analysis

Distributions of the baseline characteristics in the study cohorts 2019, 2020, and 2021 are described. Continuous variables were summarized as the mean and standard deviation and compared using an analysis of variance. Linear contrast coefficients were used to test the linear trends of HbA1c-before and HbA1c-after across years. Categorical variables are expressed as counts and percentages and were compared using the chi-square test among groups. Bonferroni’s correction rule was applied for multiple comparisons. We adapted the general estimation equation (GEE) method in the linear regression model to compare the ΔHbA1c among different study years. An autoregressive working correlation structure of the ΔHbA1c was assumed in the GEE model, as the structure minimized the value of quasi-likelihood under the independence model criterion (QIC) statistic. The regression coefficient (β) corresponded to the changes in ΔHbA1c between years, for example, the ΔHbA1c in 2019 minus ΔHbA1c in 2021. In addition to univariable analyses, multivariable regression analyses were performed to adjust for potential confounding effects from patients’ characteristics and clinical features. To appropriately consider the effects of time-varying clinical features, we collected the first data of these clinical information in each year, so that three different values in three years were all included in regression analyses. In the multivariable GEE models, we managed these clinical data as time-dependent covariates and estimated the regression coefficients after adjustment for these covariate contributions on the ΔHbA1c. Missing values of covariates have been replaced by mean values of the study cohorts. Type I error was set at 5% in all analyses. Data analyses were performed using SAS Enterprise Guide version 7.15 (SAS Institute Inc., Cary, NC, USA).

### 2.4. Subgroup Analyses

To evaluate the potential bias that may result from incomplete data compared among different study cohorts, we additionally conducted subgroup analyses. The subgroup included the patients who fulfilled the inclusion criteria mentioned above and were simultaneously enrolled in cohort-2019, cohort-2020, and cohort-2021. Therefore, each patient had a total of three paired HbA1c records obtained before and after the season in all three observation years, and their baseline data were retrieved between 1 January and 19 May 2019. In general, the data analysis procedures in this subgroup analyses were the same as those used in the major analyses. Multiple HbA1c measurements in the same patient during different seasons or years were pairwise compared using a paired t-test in subgroup analyses.

## 3. Results

**Figure 2:**
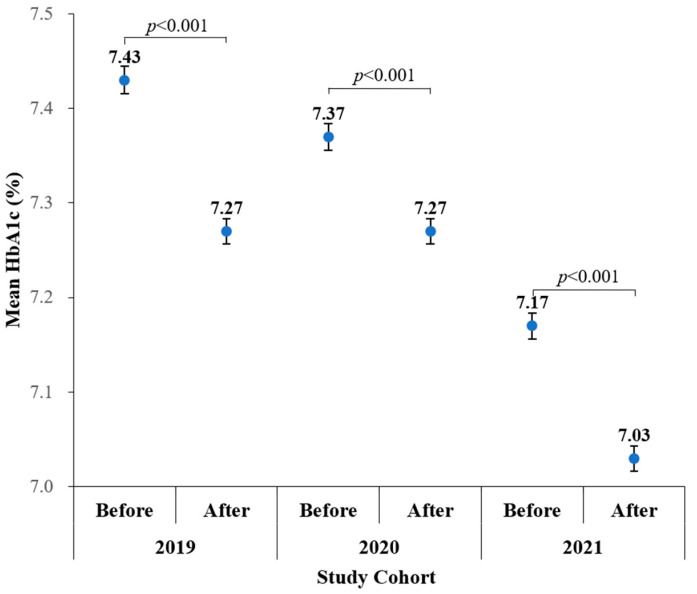
Significant changes in HbA1c levels between period-before and period-after in cohort-2019, cohort-2020, and cohort-2021, respectively. Dots represent means and error bars represent standard errors.

A total of 9111, 9078, and 8663 patients were enrolled in cohort-2019, cohort-2020, and cohort-2021, respectively. The baseline characteristics are presented in Table 1. Compared with patients in cohort-2019, those in cohort-2021 had a larger body weight, larger BMI, higher SBP, higher DBP, faster HR, lower eGFR, lower LDL, lower TG, a smaller proportion of patients with hypertension, and a larger proportion of glucagon-like peptide-1 receptor agonist (GLP-1 RA) use. Compared with patients in the cohort-2020, patients in the cohort-2021 had a smaller proportion of females, larger body height, faster HR, lower eGFR, lower TG, lower GPT, a smaller proportion of patients with hypertension, and a larger proportion of GLP-1 RA use. There were no significant differences in age and proportions of antihypertensive drugs, insulin, and oral antidiabetic drug use among the three cohorts. Comparisons of HbA1c and ΔHbA1c levels across 2019–2021 are presented in Table 2. Compared with patients in cohort-2019, those in cohort-2021 had a lower HbA1c-before and lower HbA1c-after. Compared with patients in the cohort-2020, patients in the cohort-2021 had a lower HbA1c-before, lower HbA1c-after, and lower ΔHbA1c. The values of HbA1c-before and HbA1c-after significantly decreased across the three cohorts. Figure 2 depicts that HbA1c-after was lower than HbA1c-before in 2019 (7.27 ± 1.27% vs 7.43 ± 1.38%, *p* < 0.001), 2020 (7.27 ± 1.28% vs 7.37 ± 1.34%, *p* < 0.001), and 2021 (7.03 ± 1.22% vs 7.17 ± 1.29%, *p* < 0.001).

Table 3 shows results that examined the year effect on the ΔHbA1c. Compared with cohort-2021, the values of ΔHbA1c were lower in cohort-2019 and higher in cohort-2020 in the univariable regression analysis. However, with adjustment for potential confounding effect of baseline characteristics including demographic features, body mass index, lipid profile, blood pressure, renal function, liver function, antihypertensive and hypoglycemic agents, the difference in ΔHbA1c between cohort-2020 and cohort-2021 was largely minimized and became insignificant. The decrement in ΔHbA1c during cohort-2019 was more obvious than that during cohort-2021 (β [95% confidence interval (CI)]: −0.047% [−0.073% to −0.021%], *p* < 0.001), but there was no significant difference between the ΔHbA1c in cohort-2020 and cohort-2021 (β [95% CI]: 0.009% [−0.017% to 0.035%], *p* = 0.500) after adjustment for baseline characteristics. Moreover, several factors also affected their seasonal changes in HbA1c. Patients with TG ≥ 150 mg/dL, SBP ≥ 140 mmHg, eGFR < 60 mL/min/1.73 m^2^, high GPT, and use of antihypertensive drugs had a significantly high value of ΔHbA1c; however, patients receiving insulin and OHA treatment had a significantly low value of ΔHbA1c.

Table 4 shows baseline characteristics of 3720 subjects included in subgroup analyses. Compared to the patients in the main analyses, the subgroup had similar distributions in age, sex, anthropometry, and blood pressure, and slightly lower TG and LDL levels. However, their eGFR values were similar to cohort-2019 and cohort-2020, but higher than cohort-2021; the proportions of receiving oral hypoglycemic agent (OHA) and GLP-1 RA treatments were higher than that in all three cohorts in the main analyses. The HbA1c-before, HbA1c-after, and ΔHbA1c values from different seasons in this subgroup were very close to that in the main analyses (Table 5). Compared to 2021, the HbA1c-before and HbA1c-after were higher in 2019 and 2020. There was a significant decreasing trend for HbA1c-before (*p* < 0.001) and HbA1c-after (*p* < 0.001) across the three years. The decrements in ΔHbA1c were more obvious in 2019 than in 2021 (β [95% CI]: −0.037% [−0.068% to −0.006%], *p* = 0.019), while not different between 2020 and 2021 (*p* = 0.156). The linear regression analyses using the GEE method shows consistent results (Table 6).

## 4. Discussion

The main finding of our study was that the ΔHbA1c values were not significantly different between 2020 (Lv2 alert) and 2021 (Lv3 alert), even though the HbA1c-after values were significantly lower than the HbA1c-before values in the same observation year. Another finding was a significantly lower ΔHbA1c in 2019 (without COVID-19) compared to 2021. The findings of the subgroup analyses were consistent with those of the main analyses. The present study indicated the absolute seasonal reduction in HbA1c was slightly lessen during the years with COVID-19 than the year without COVID-19. However, there was no deterioration in HbA1c level after the lockdown (raising the COVID-19 alert from Lv2 to Lv3) during the years with COVID-19.

We found reductions in the HbA1c levels after the Lv3 alert in 2021, but there was a similar reduced HbA1c levels between the study seasons in the previous two years. Seasonal variations in glycemic control may explain this finding. Many previous studies have shown that HbA1c is higher in cold seasons than in warm seasons [16,17,18], and this variation might be associated with increased dietary intake and lower physical activity in colder seasons [26,27,28]. Cultural events may also influence seasonal variability in HbA1c levels. A prospective study in Taiwan reported a poor glycemic control during the Chinese New Year holiday [29], it was very likely due to the potentially increased calorie consumption. Traditionally, the Chinese population partake in elaborate dinners and consume large amounts of candies and snacks in family reunion or friends visits during the entire holiday period. In 2019 and 2021, the Chinese New Year holiday was in early February and the end of January, just around the beginning of the season-before defined in our study. HbA1c represents the integrated glucose concentration during the preceding 8–12 weeks [30]; therefore, higher HbA1c levels over the period before in the present study might be affected by lifestyle changes during the Chinese New Year holiday. A previous study that did not explore the impact of the lockdown revealed that the COVID-19 pandemic did not influence the pattern of seasonal variation in HbA1c levels [31]. This is in agreement with our finding of an unchanged pattern of seasonal variation of HbA1c during the COVID-19 pandemic. Despite the pattern of seasonal variation of HbA1c being unchanged, we found a slightly smaller reduction in the HbA1c values from the season-before to season-after during the years with COVID-19 than during the year without COVID-19.

Many studies have explored the effects of COVID-19-associated lockdown measures on glycemic control, but the findings have been inconsistent. For instance, worsening glycemic control was reported in India [32], China [33], and Korea [34], respectively. In contrast, a study from Greece [35] and another study from India [36] showed improved glycemic control during the COVID-19 pandemic. Moreover, in studies conducted in Italy and Turkey, glycemic control did not change significantly [37,38]. Heterogeneous results have also been demonstrated in meta-analyses. Silverii et al. [11] found no significant change in the HbA1c levels, but Ojo et al. [13] revealed a significant increase in HbA1c and fasting glucose levels following a lockdown due to COVID-19. Different study designs and geographic variations in the countries where the studies were conducted might explain the heterogeneity in the findings. The strengths of restriction measures may also differ from country to country. For example, the restriction was particularly strict in Italy and Spain compared other countries [11]. In general, the restriction was less strict in some Asia countries, such as Japan, South Korea, and Taiwan, than in Europe. A subgroup analysis in Silverii’s study also revealed a reduced HbA1c levels in Asia but no significant changes in HbA1c in Europe [11].

Notably, the confounding effect of seasonal glycemic change has not been appropriately managed in most previous studies. However, in contrast to our findings, a study conducted in East Asia and South Korea showed a lack of seasonal variation in HbA1c levels and increased HbA1c levels following enhanced social distancing during the COVID-19 outbreak [34]. The worsening glycemic control has been explained by some studies, which demonstrated changes in lifestyle during the COVID-19 lockdown, including lack of physical activities, increased dietary intake, more screen time, and weight gain [6,7,8]. Stress and anxiety might mediate adverse lifestyle changes and poor glycemic control during the COVID-19 lockdown [8]. However, it is noteworthy that, in another study, increased levels of perceived stress and less exercise during the COVID-19 lockdown did not lead to a deterioration of glycemic control [39]. On the other hand, the messages alerting diabetes as a risk factor in developing critical situations of COVID-19 have been widely disseminated; therefore, patients with diabetes may pay more attention to self-manage their glucose levels and better adhere to medications during lockdown.

The present study showed no significant difference in ΔHbA1c levels between the cohorts in 2020 and 2021. Briefly, lockdown measures (raising the COVID-19 alert from Lv2 to Lv3) did not lead to the deterioration of glycemic control. During the period of the Lv3 alert in Taiwan, hospital capacity was relatively adequate in central Taiwan. Many countries reported that COVID-19 had disrupted the care of people with diabetes [3]. In contrast, the national health insurance system, with coverage of more than 99% of citizens in Taiwan [22] and medical access for diabetic management, was not disrupted. Furthermore, drive-through services to patients with chronic illness to refill prescriptions, which might increase the accessibility of prescription drugs and largely avoid the risk of contact transmission, were implemented. The impact of lockdown measures on glycemic control in patients with type 2 DM may not be significant. However, the extent to which lifestyle changes might affect glycemic control during the relatively short duration (69 days) of the Lv3 alert in Taiwan needs to be empirically examined.

In addition to the effect of the lockdown, we found a downward trend in HbA1c levels across years in the present study. Different from our results, an elevated mean HbA1c level was observed in Japan in 2020, the year when the COVID-19 pandemic was most prevalent, compared with the previous two years [31]. However, in line with our findings, previous studies evaluating the quality of diabetic control in Taiwan have demonstrated a downward trend in HbA1c levels across more than a decade [40,41,42]. The annual HbA1c improvement might result from the pay-for-performance program for diabetes shared care in Taiwan [43]. The downward trend of HbA1c in our study was likely to be attributed to improved HbA1c control following the improved standard of diabetic care. Furthermore, it appears that the influence of the COVID-19 pandemic might be minimal in Taiwan. However, the impact of the COVID-19 pandemic on year-to-year variation in glycemic control needs to be explored in a longer-term study.

Another finding in the present study was that weight and BMI increased compared to the year without the COVID-19 pandemic, even through the prevalence of insulin and OHA use did not change. Several previous studies revealed weight gains in patients with diabetes during the COVID-19 pandemic. Worsening weight control was found to be associated with adverse lifestyle changes including decreased physical activity [6,7,8,43], increased frequency of snack eating, and carbohydrate consumption [7,8]. On the other hand, some studies also found that increased fear and stress during the COVID-19 pandemic might be associated with adverse lifestyle change [8,39]. According to a previous study to investigate lifestyle changes during the COVID-19 Lv3 alert in the Taiwan general population, the online survey showed a significant decrease in physical activity but no significant changes in body weight during this period [44]. Notably, it has been reported that an increasing trend in obesity across more than a decade based on the data from Nutrition and Health survey in Taiwan [45]. However, further studies focusing on patients with diabetes to explore weight and lifestyle changes between the years with and without the COVID-19 pandemic in Taiwan are needed.

This study had several strengths. The assessment of the impact of COVID-19 lockdown on glycemic control was not simply based on the before-and-after comparison, we took the seasonal variation of HbA1c levels into account, and compared the ΔHbA1c values between the year without COVID-19, the year with COVID-19 but not lockdown (Lv2 alert), and the year with COVID-19 lockdown (Lv3 alert). We also adjusted for other demographical and clinical baseline characteristics of patients using GEE model to appropriately consider the dependency of multiple measures of HbA1c. Lastly, we additionally conducted a subgroup analysis for patients with complete follow-up, demonstrating similar results with main analyses, and increasing the robustness of our conclusions.

The present study also had several limitations. First, only HbA1c levels were analyzed to assess blood glucose control in patients with type 2 DM. Second, this was a single-center retrospective study in central Taiwan, and our results might not be representative of the entire patient population. Third, a definite causal relationship between the Lv3 alert and glycemic control could not be established based on an observational design. Fourth, glycemic control is significantly affected by patients’ lifestyle. However, we did not have the information of lifestyle change during the COVID-19 pandemic in this retrospective cohort study. Fifth, we did not collect the dosages and categories of antidiabetic drugs in the present study. Finally, there could be selection bias because some patients were lost to follow-up and lacked complete HbA1c data during the COVID-19 pandemic. The situations of patients remaining in the follow-up may tend to be more complicated or severe. However, in the subgroup analyses, we additionally included patients with complete follow-up and performed a subgroup analysis, the consistent results between subgroup analysis and main analysis suggested robust observations in our study.

## 5. Conclusions

Compared with the HbA1c levels in season-before, HbA1c levels in season-after significantly decreased in all three years. However, there was no significant difference in seasonal reduction in HbA1c between the year with COVID-19 pandemic without lockdown (2020) and the year with COVID-19 pandemic with lockdown (2021). The absolute value of seasonal HbA1c reduction was slightly lessen during the COVID-19 pandemic compared to the year without COVID-19 (2019) in the present study. The long-term studies for time-series analyses are warranted to clarify the impact of COVID-19 pandemic on the seasonal glycemic variation.

## Figures and Tables

**Table 1:** Baseline characteristics of the enrolled patients in three study cohorts.

Baseline Characteristics	Cohort-2019	Cohort-2020	Cohort-2021	*p* ^†^
	(*n* = 9111)	(*n* = 9078)	(*n* = 8663)	
Age (years)	65.3 ± 12.6	65.0 ± 12.4	64.9 ± 12.2	0.154
Female, *n* (%)	4109 (45.1)	4044 (44.5) *	3734 (43.1)	0.022
Height (cm)	162.0 ± 6.4	161.9 ± 7.4 *	162.2 ± 7.2	0.012
Weight (kg)	68.2 ± 10.2 *	69.0 ± 12.0	69.3 ± 11.5	<0.001
BMI (kg/m^2^)	26.0 ± 3.2 *	26.3 ± 3.8	26.3 ± 3.6	<0.001
SBP (mmHg)	131.8 ± 14.1 *	133.8 ± 16.9	134.0 ± 15.4	<0.001
DBP (mmHg)	71.9 ± 9.5 *	73.4 ± 11.2	73.3 ± 9.7	<0.001
HR (beat/min)	81.9 ± 10.1 *	82.9 ± 11.9 *	84.0 ± 10.8	<0.001
eGFR (mL/min/1.73 m^2^)	71.08 ± 25.76 *	71.03 ± 25.32 *	65.81 ± 22.88	<0.001
LDL cholesterol (mg/dL)	87.0 ± 22.3 *	85.8 ± 21.5	85.1 ± 22.1	<0.001
TG (mg/dL)	151.2 ± 101.2 *	154.9 ± 112.5 *	147.1 ± 106.9	<0.001
GPT (U/L)	26.8 ± 18.5	27.2 ± 18.6 *	26.3 ± 16.4	<0.001
Hypertension, *n* (%)	6555 (71.9) *	6458 (71.1) *	5609 (64.7)	0.003
Anti-hypertensive drug use, *n* (%)	5724 (62.8)	5696 (62.7)	5423 (62.6)	0.952
Insulin use, *n* (%)	1875 (20.6)	1843 (20.3)	1721 (19.9)	0.492
OHA use, *n* (%)	7957 (87.3)	7942 (87.5)	7599 (87.7)	0.739
GLP-1 RA use, *n* (%)	445 (4.9) *	449 (4.9) *	502 (5.8)	0.010

Continuous data are expressed as means ± standard deviation and categorical data are expressed as numbers (percentages); * indicates *p* < 0.05 compared to cohort-2021. ^†^ indicates *p* value for linear trend. BMI, body-mass index; DBP, diastolic blood pressure; eGFR, estimated glomerular filtration rate; GLP-1 RA, glucagon-like peptide-1 receptor agonist; GPT, glutamic pyruvic transaminase; HR, heart rate; LDL, low-density lipoprotein; OHA, oral hypoglycemic agent; SBP, systolic blood pressure; TG, triglycerides.

**Table 2:** Comparisons of HbA1c and ΔHbA1c levels across 2019–2021.

Variables	Cohort-2019	Cohort-2020	Cohort-2021	*p* ^†^
	(*n* = 9111)	(*n* = 9078)	(*n* = 8663)	
HbA1c-before (%)	7.43 ± 1.38 *	7.37 ± 1.34 *	7.17 ± 1.29	<0.001 ^†^
HbA1c-after (%)	7.27 ± 1.27 *	7.27 ± 1.28 *	7.03 ± 1.22	<0.001 ^†^
ΔHbA1c (%)	−0.16 ± 0.83	−0.10 ± 0.83 *	−0.14 ± 0.76	0.001

Continuous data are expressed as means ± standard deviation; * indicates *p* < 0.05 compared to cohort-2021. ^†^ indicates *p* value for linear trend. HbA1c, hemoglobin A1c.

**Table 3:** Multivariable linear regression analyses using generalized estimation equation (GEE) to examine associates with ΔHbA1c values.

Parameters	Unit of *β*	Univariable Analysis			Multivariable Analysis		
		*β*	95% CI	*p*	*β*	95% CI	*p*
Year	Cohort-2019 vs. 2021	−0.024	−0.048, −0.001	0.040	−0.047	−0.073, −0.021	<0.001
	Cohort-2020 vs. 2021	0.035	0.011, 0.058	0.004	0.009	−0.017, 0.035	0.500
Age	per 10 years increase				0.008	−0.003, 0.018	0.138
Sex	Male vs. Female				−0.041	−0.061, −0.02	<0.001
BMI	≥24 vs. <24 (kg/m^2^)				0.014	−0.011, 0.039	0.264
LDL cholesterol	≥100 vs. <100 (mg/dL)				−0.020	−0.052, 0.012	0.224
TG	≥150 vs. <150 (mg/dL)				0.050	0.028, 0.072	<0.001
SBP	≥140 vs. <140 (mmHg)				0.036	0.013, 0.060	0.003
eGFR	<60 vs ≥60 (mL/min/1.73 m^2^)				0.029	0.003, 0.055	0.027
GPT	per 10 U/L increase				0.017	0.010, 0.023	<0.001
HR	per 10 beat/min increase				−0.001	−0.010, 0.008	0.851
Antihypertensive drug	Use vs. no				0.043	0.021, 0.065	<0.001
Insulin	Use vs. no				−0.120	−0.154, −0.087	<0.001
OHA	Use vs. no				−0.083	−0.110, −0.055	<0.001
GLP-1 RA	Use vs. no				0.023	−0.037, 0.083	0.456

Dependent variables: ΔHbA1c; β, regression coefficient in linear regression. CI, confidence interval. BMI, body-mass index; eGFR, estimated glomerular filtration rate; GLP-1 RA, glucagon-like peptide-1 receptor agonist; GPT, glutamic pyruvic transaminase; HbA1c, hemoglobin A1c; HR, heart rate; LDL, low-density lipoprotein; OHA, oral hypoglycemic agent; SBP, systolic blood pressure; TG, triglycerides.

**Table 4:** Baseline characteristics of the enrolled patients with paired HbA1c in all three years.

Basic Characteristics	Total (*n* = 3720)
Age (years)	63.9 ± 11.8
Female, *n* (%)	1687 (45.4)
Height (cm)	162.0 ± 9.0
Weight (kg)	69.9 ± 14.6
BMI (kg/m^2^)	26.5 ± 4.59
SBP (mmHg)	136.1 ± 19.4
DBP (mmHg)	73.8 ± 12.0
HR (beat/min)	84.5 ± 13.2
eGFR (mL/min/1.73 m^2^)	70.92 ± 24.71
LDL cholesterol (mg/dL)	84.06 ± 26.63
TG (mg/dL)	133.5 ± 109.2
GPT (U/L)	24.9 ± 16.4
Hypertension, *n* (%)	2547 (68.5)
Anti-hypertensive drug use, *n* (%)	2407 (64.7)
Insulin use, *n* (%)	804 (21.6)
OHA use, *n* (%)	3504 (94.2)
GLP-1 RA use, *n* (%)	325 (8.7)

Digits represent mean ± standard deviation and categorical data are expressed as numbers (percentages). BMI, body-mass index; DBP, diastolic blood pressure; eGFR, estimated glomerular filtration rate; GLP-1 RA, glucagon-like peptide-1 receptor agonist; GPT, glutamic pyruvic transaminase; HbA1c, hemoglobin A1c; HR, heart rate; LDL, low-density lipoprotein; OHA, oral hypoglycemic agent; SBP, systolic blood pressure; TG, triglycerides.

**Table 5:** Comparisons of HbA1c and ΔHbA1c levels across 2019–2021 in the subgroup with complete HbA1c measurements in all three years.

Variables	Year (*n* = 3720)			
	2019	2020	2021	*p*-For-Trend ^†^
HbA1c-before (%)	7.41 ± 1.24 *	7.37 ± 1.20 *	7.18 ± 1.17	<0.001
HbA1c-after (%)	7.27 ± 1.16 *	7.28 ± 1.14 *	7.07 ± 1.13	<0.001
ΔHbA1c (%)	−0.15 ± 0.73 *	−0.09 ± 0.68	−0.11 ± 0.66	0.034

Data are expressed as means ± standard deviation; * indicates *p* < 0.05 compared with that in 2021. ^†^ *p* was estimated using linear regression with GEE method. HbA1c, hemoglobin A1c.

**Table 6:** Linear regression analyses using generalized estimation equation for ΔHbA1c only in patients with paired HbA1c in all three years.

ΔHbA1c (%)	Univariable Analysis		
	*β*	95% CI	*p*
Cohort-2019 vs. 2021	−0.037	−0.068, −0.006	0.019
Cohort-2020 vs. 2021	0.022	−0.008, 0.051	0.156

Dependent variables: ΔHbA1c; *β*, regression coefficient in linear regression. CI, confidence interval. HbA1c, hemoglobin A1c.

## Data Availability

The data presented in this study are available on request from the corresponding author.
